# Tau Protein Aggregation Inhibitors—Therapeutic Strategy for Concurrent Tau and Amyloid Aggregation Inhibition

**DOI:** 10.3390/biomedicines14030522

**Published:** 2026-02-26

**Authors:** Thomas Gabriel Schreiner, Romeo Cristian Ciobanu, Oliver Daniel Schreiner

**Affiliations:** 1 Department of Medical Specialties III, Faculty of Medicine, “Grigore T. Popa” University of Medicine and Pharmacy, 700115 Iasi, Romania; schreiner.oliver-daniel@d.umfiasi.ro; 2 First Neurology Clinic, “N. Oblu” Clinical Emergency Hospital, 700309 Iasi, Romania; 3 Department of Electrical Measurements and Materials, Gheorghe Asachi Technical University, 700050 Iasi, Romania; 4 Medical Oncology Department, Regional Institute of Oncology, 700483 Iasi, Romania

**Keywords:** tau protein, Alzheimer’s disease, neurodegeneration, small-molecule inhibitor, curcumin

## Abstract

Tau protein, a microtubule-associated protein widely distributed in the central nervous system, aggregates abnormally and forms neurofibrillary tangles in neurodegenerative diseases. Particularly in Alzheimer’s disease, pathological tau protein aggregates disrupt the structure and function of neurons, triggering other neurodegenerative-related processes such as neuroinflammation and amyloid plaque formation, and finally leading to neuronal death. Several classes of drugs targeting neurofibrillary tangles have recently been studied, with tau protein aggregation inhibitors as a key research direction. In the context of emerging therapeutic perspectives, this review aims to provide an updated, practical overview of currently available tau protein aggregation inhibitors and future research directions. The first part of the manuscript highlights the pathophysiological basics of tau protein aggregation and tau-related changes in neurodegenerative disorders, with a focus on Alzheimer’s disease pathology. Subsequently, the most relevant classes of drugs that inhibit tau protein aggregation, including small-molecule inhibitors and natural compounds, are presented, with examples from recent clinical trials. Finally, beyond summarizing established classes of tau aggregation inhibitors, this review places particular emphasis on emerging and comparatively underexplored compounds with dual activity against both tau and amyloid-β pathology. The originality and novelty of this work arise from the systematical analysis of recent preclinical and clinical evidence with a translational, practice-oriented perspective, highlighting mechanistic convergence, repurposing opportunities, and therapeutic combinations that may better reflect the multifactorial nature of neurodegenerative diseases. Thus, this work provides a forward-looking framework for future drug development and identifies promising candidates that may shape the next generation of disease-modifying therapies.

## 1. Introduction

Tau protein is a microtubule-associated protein predominantly expressed in neurons, where it plays a central role in stabilizing microtubules and supporting axonal transport [1]. Under physiological conditions, tau undergoes a finely regulated pattern of post-translational modifications, with phosphorylation being the most notable one, that modulate its binding affinity for microtubules [2]. Dysregulation of these processes leads to conformational changes in the protein, reduced microtubule affinity, increased tau misfolding, and the final formation of insoluble aggregates [3]. These aggregates encompass a spectrum of mainly intracellularly concentrated abnormal species, including oligomers, paired helical filaments, and neurofibrillary tangles (NFTs) [4]. The transition from soluble monomeric tau to pathogenic insoluble assemblies is a multifactorial process influenced by genetic and environmental factors, with oxidative stress [5], chronic neuroinflammation [6], and interactions with other abnormal proteins, such as amyloid beta (Aβ) [7], as relevant associated processes. Because of its central role in maintaining cytoskeletal integrity and neuronal function, perturbations in tau homeostasis have profound consequences for neuronal viability [8].

**Figure 1:**
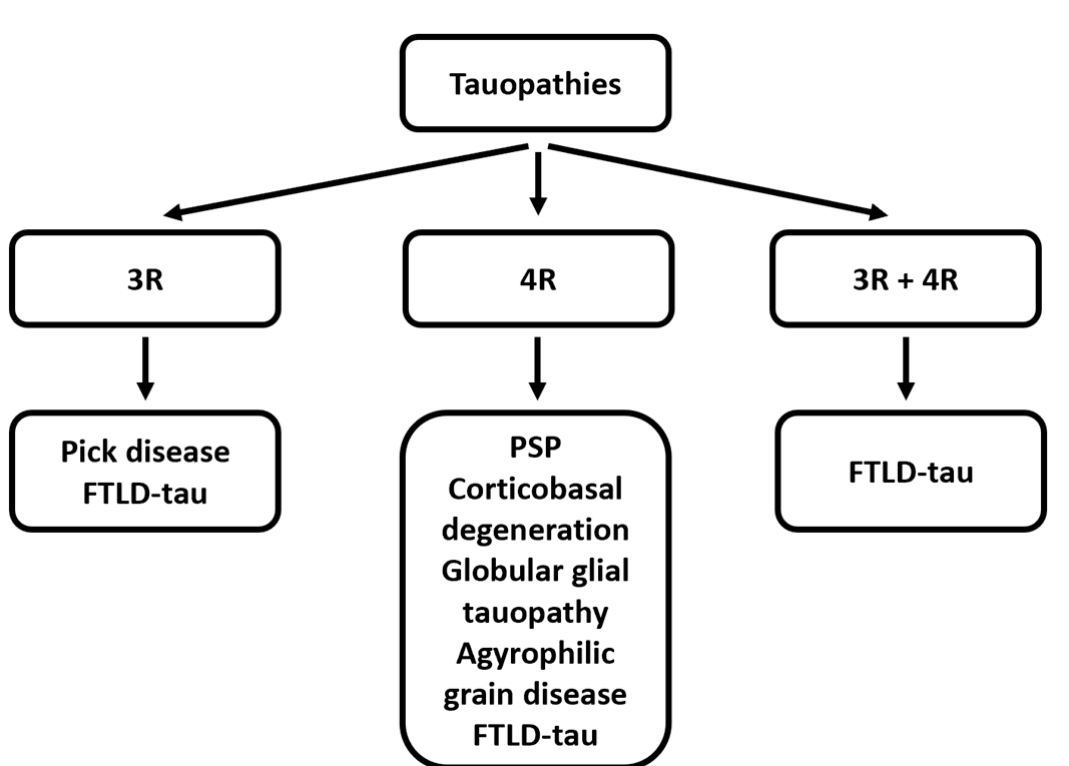
Classification of tauopathies according to pathocal tau aggregations of isoforms with 3 repeats (3R), 4 repeats (4R) of the microtubule binding domain, or a mixture of 3R and 4R isoforms (PSP—progressive supranuclear palsy; FTLD—frontotemporal lobar degeneration).

Aberrant tau aggregation is a defining hallmark of a heterogeneous subgroup of neurodegenerative diseases collectively known as tauopathies (Figure 1). These include Alzheimer’s disease (AD), frontotemporal lobar degeneration with tau pathology (FTLD-tau), progressive supranuclear palsy (PSP), and corticobasal degeneration (CBD) [9]. In these disorders, tau pathology correlates strongly with synaptic dysfunction, neuronal loss, and clinical progression [10]. In AD in particular, the burden and anatomical spread of tau pathology show a close relationship to cognitive decline, underscoring tau’s pivotal role in disease mechanisms [11]. Pathogenic tau species are thought to impair axonal transport, disrupt synaptic signaling, perturb mitochondrial function, and propagate trans-synaptically in a prion-like fashion, facilitating the spread of pathology across neural networks [12]. Consequently, tau has emerged as a critical therapeutic target, with great potential to modify disease onset or slow its progression by preventing the formation, accumulation, or propagation of toxic tau species [13].

Currently approved pharmacological treatments for AD remain largely symptomatic. Acetylcholinesterase (AChE) inhibitors, such as donepezil, rivastigmine, and galantamine, enhance cholinergic neurotransmission and provide modest, temporary cognitive benefits, while the NMDA receptor antagonist memantine targets glutamatergic excitotoxicity. More recently, anti-amyloid monoclonal antibodies have aimed to modify disease progression by reducing Aβ burden; however, their clinical benefits remain moderate and are accompanied by safety and implementation challenges. Importantly, none of these approaches directly targets tau aggregation, which correlates more closely with cognitive decline and disease severity than amyloid burden. The limited disease-modifying efficacy of current therapies underscores the need for alternative or complementary strategies, including tau-directed interventions such as tau aggregation inhibitors.

Currently, the tau-targeting therapeutic landscape includes multiple strategies that modulate tau biology. Tau-targeting approaches comprise four broad categories: kinase and phosphatase modulators designed to correct aberrant phosphorylation; microtubule stabilizers that compensate for tau loss of function; immunotherapies that neutralize extracellular or intracellular tau species; and agents that directly prevent the misfolding, oligomerization, or fibrillization of tau [14]. Within this framework, tau protein aggregation inhibitors occupy a distinct and highly promising niche. Rather than modulating upstream regulatory pathways or enhancing downstream compensatory mechanisms, these compounds aim to intervene at the core pathogenic event, mainly the structural conversion of tau into pathological aggregates [15]. By inhibiting nucleation, elongation, and fibril stabilization, tau aggregation inhibitors aim to preserve the physiological pool of soluble tau, reduce the formation of toxic oligomeric intermediates, and ultimately limit the spread of tau pathology [16]. Various molecules, including polyphenols, benzothiazoles, rhodanines, anthraquinones, and novel small molecules, have shown potential to inhibit tau aggregation in vitro and in preclinical models [17]. As the field evolves, tau aggregation inhibitors increasingly complement and, in some cases, may synergize with immunotherapeutic and phosphorylation-modulating strategies. Their mechanistic specificity makes them an attractive category for disease-modifying interventions, particularly as our understanding of tau structural dynamics and aggregation pathways continues to advance.

In the context of significant advances in the field in recent years, this review aims to provide an updated, clinically oriented synthesis of tau protein aggregation inhibitors, including small-molecule inhibitors and natural compounds, with examples from recent clinical and preclinical trials. While several recent reviews have comprehensively cataloged tau-targeting agents or focused predominantly on mechanistic aspects of aggregation, the present work aims to connect structural and pathophysiological insights with pharmacological and translational considerations. Particular emphasis is placed on comparatively understudied or emerging compounds, including repurposed drugs and molecules with potential dual activity against both tau and amyloid-β pathology, analyzed within a therapeutic development framework. By integrating mechanistic background with recent preclinical and clinical data, this review aims to provide a practice-oriented perspective that highlights translational relevance and identifies strategic directions for future drug development.

## 2. Impact of Tau Protein on Neurodegenerative Diseases

### 2.1. Role of Tau Protein in Physiological Conditions

Tau protein, encoded by the MAPT gene on chromosome 17q21.31, is a microtubule-associated protein essential for microtubule stabilization, predominantly expressed in cortical and hippocampal neurons but also present in glial cells, the peripheral nervous system, and extracellular fluids such as the cerebrospinal fluid (CSF) [18]. Structurally, tau comprises N- and C-terminal domains, a proline-rich region targeted by kinases, and a microtubule-binding domain whose interactions are modulated by phosphorylation [19]. The MAPT gene contains 16 exons and undergoes complex, developmentally regulated alternative splicing that produces six major isoforms in the adult human brain, defined by the presence or absence of N-terminal inserts (0N, 1N, 2N) and by three or four microtubule-binding repeats (3R, 4R) [20]. Isoform expression patterns shift during neurodevelop-ment: early brains predominantly express 0N3R, whereas mature brains maintain a balanced 3R/4R ratio. These dynamic splicing changes, particularly in exons 2 and 10, may be critical for neuronal maturation and are implicated in tau-related neuropathology [21].

**Figure 2:**
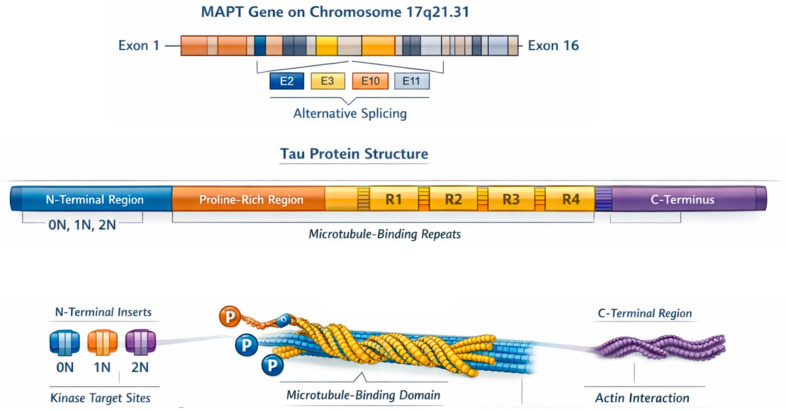
Tau protein relevant structural aspects: a product of the MAPT gene, N-terminal relevant inserts, and interactions with other structures of the neuronal cytoskeleton.

Tau protein is a central regulator of the neuronal cytoskeleton, coordinating microtubule stability, actin dynamics, and axonal transport. As a key microtubule-associated protein, tau supports neuronal morphology, neurite formation, and axonal pathfinding, with its expression and phosphorylation patterns tightly regulated during development [22]. The C-terminal microtubule-binding region and the proline-rich domain mediate tau’s interactions with both microtubules and filamentous actin, enabling actin bundling and influencing dendritic structure and synaptic integrity [23] (Figure 2). These highly dynamic, multivalent interactions rely on multiple actin-binding sites within tau, although the precise mechanisms of actin–tau–microtubule cross-linking remain incompletely understood.

Tau also modulates fast axonal transport by differentially influencing kinesin and dynein motor proteins; this regulatory role is phosphorylation-dependent and is disrupted by pathological kinase activity such as GSK3β overactivation in Alzheimer’s disease [24]. Beyond its cytoplasmic functions, tau contributes to synaptic signaling through its interaction with Fyn kinase and participates in myelination processes in oligodendrocytes [25]. In the nucleus, tau binds to DNA and protects genomic integrity under oxidative stress [26]. Finally, physiological tau is actively released into the extracellular space in an activity-dependent manner, where neighboring neurons can take it up and seed further tau pathology. Enhanced neuronal activity not only increases extracellular tau availability but also accelerates its transneuronal propagation and pathological accumulation in vivo. This activity-driven extracellular transfer of tau provides a mechanistic link between neuronal hyperexcitability and the progressive spread of tauopathy in Alzheimer’s disease, highlighting neuronal activity and tau release pathways as potential therapeutic targets [27]. Collectively, tau’s diverse structural and functional roles underscore its essential role in neuronal maintenance and explain its significant impact in neurodegenerative diseases. Table 1 summarizes the tau protein’s functions in physiological conditions.

### 2.2. Tau Alterations and Impact on Neurodegenerative Diseases

Despite tau protein playing important roles in physiological conditions, the historical and molecular characterization of tau-related pathology has established tau as a central driver of neurodegeneration [28]. Following Alois Alzheimer’s first description of neurofibrillary tangles (NFTs) in 1906, subsequent studies identified these inclusions as paired helical filaments (PHFs) composed predominantly of hyperphosphorylated tau [29]. Advances in structural and biochemical analyses have revealed that tau aggregates differ markedly across tauopathies, with disease-specific filament conformations and isoform compositions contributing to clinical and pathological heterogeneity. Cryo-electron microscopy showed that paired-helical filaments (PHFs) and straight filaments (SFs) purified from AD brains contain a core formed by residues ~306–378 of tau arranged in a combined cross-β/β-helix conformation; AD filaments contain both 3R and 4R tau isoforms [30]. Filaments in Pick’s disease have a distinct, three-lobed fold that is composed exclusively of 3R tau and is topologically and chemically different from the AD fold, demonstrating that identical primary sequences can adopt alternative, disease-specific conformations [31]. CBD and other 4R-predominant tauopathies also display unique filament folds: cryo-EM structures from CBD show a novel fold and protofilament arrangement that is consistently different from both AD and Pick’s disease and is dominated by 4R tau isoforms [32]. PSP and related 4R disorders likewise possess characteristic filament architectures; systematic cryo-EM studies across multiple tauopathies support a structure-based classification in which the identity (3R vs. 4R vs. mixed) and the precise protofilament fold correlate with neuropathological phenotype [33].

Tau undergoes numerous post-translational modifications (PTMs), of which phosphorylation is the most extensively implicated in pathogenesis [34]. Hyperphosphorylation occurs at key serine, threonine, and tyrosine residues, most prominently S202/T205, T231, S214, S262, S356, S396/S404, S422, and Y18, several located within or adjacent to the microtubule-binding domain, where they disrupt tau–microtubule interactions and promote aggregation [35]. This reduced microtubule affinity facilitates tau dissociation and the progressive formation of dimers, oligomers, paired helical filaments (PHFs), and ultimately neurofibrillary tangles (NFTs) [36]. Dysregulated kinase activity (e.g., CDK5, GSK3β, JNK) combined with impaired dephosphorylation by phosphatase PP2A underlies these pathogenic phosphorylation patterns [37].

**Figure 3:**
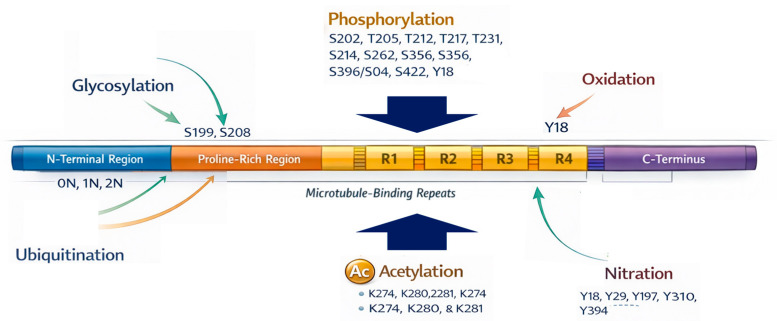
Schematic representation of post-translational modifications of tau protein.

Beyond phosphorylation, tau is modified by glycosylation, ubiquitination, acetylation, nitration, truncation, methylation, and oxidative changes, several of which further modulate aggregation propensity [38]. N-glycosylation may precede phosphorylation, promoting a conformation more susceptible to hyperphosphorylation and aggregation, while also impairing microtubule binding and enhancing PHF assembly [39]. In contrast, O-GlcNAcylation appears protective: reduced O-GlcNAc levels correlate with increased tau phosphorylation and aggregation, whereas enhancement of this modification ameliorates tauopathy phenotypes in experimental models [40]. Ubiquitination, typically occurring at lysine residues within the microtubule-binding region or C-terminal tail, reflects both a clearance attempt and a contributor to aggregate maturation when proteostasis mechanisms are overwhelmed [41]. Acetylation of lysine residues, particularly K274, K280, and K281, reduces microtubule affinity, promotes cytosolic accumulation of aggregation-prone tau, and interferes with ubiquitin-mediated degradation by masking lysine residues [41,42]. Nitration (e.g., Y18, Y29) disrupts tau–Fyn interactions and synaptic signaling, while nitration within the repeat domain impairs microtubule stabilization and accelerates PHF formation [43]. Truncation events, especially C-terminal cleavage at D421, promote rapid PHF assembly and neurotoxicity, whereas N-terminal truncations expose aggregation-prone motifs and enhance seeding activity [44]. Finally, oxidation of tyrosine residues (Y18, Y197, Y310, Y394) contributes to oxidative stress–induced dityrosine cross-links that stabilize aggregation-prone tau species [45] (Figure 3).

More recently, tau PTMs, particularly phosphorylation and acetylation, have been shown to promote liquid–liquid phase separation (LLPS), leading to dense tau-rich droplets that can accelerate fibrillization and toxicity [46]. Together, these mechanistic insights underscore the complexity of tau aggregation and highlight PTM-driven structural diversification as a key determinant of tauopathy pathogenesis.

### 2.3. Tau Protein as a Key Player in Neurodegeneration—Links to Other Relevant Disease Mechanisms

Tau pathology comprises several structural and functional modifications, as detailed in the previous section; however, its complexity and pathological impact are mediated by a bidirectional network of molecular interactions that include amyloid-β (Aβ) pathology, neuroinflammatory cascades, and oxidative stress. Understanding these interconnected mechanisms is crucial for understanding the onset and evolution of neurodegeneration and for identifying more efficient, disease-modifying therapies.

The amyloid cascade hypothesis initially positioned Aβ as the initiating event in AD [47], with tau pathology emerging downstream. However, contemporary findings support a bidirectional Aβ–tau interaction model, in which each pathology amplifies the toxicity of the other [48]. Soluble Aβ oligomers induce tau hyperphosphorylation through kinases such as GSK-3β, CDK5, and MAPK, leading to tau mislocalization from axons to somatodendritic compartments [49]. This mislocalization impairs microtubule stability, disrupts axonal transport, and predisposes tau to form oligomers and fibrils [50]. Experimental models reveal that Aβ burden accelerates tau seeding and spreading, including trans-synaptic propagation of pathological tau species [51]. Conversely, tau mediates the neurotoxic effects of Aβ; for instance, Aβ-induced synaptic dysfunction depends on postsynaptic tau and its interactions with Fyn kinase, which modulates NMDA receptor excitotoxicity [52]. Removing tau, or blocking its dendritic mislocalization, attenuates Aβ-driven synaptic impairment [53].

Aβ pathology also shapes tau strain diversity. Distinct Aβ species can influence tau filament conformations, which may underlie the heterogeneous clinical presentations of AD. Although monomeric Aβ itself is essentially non-toxic, soluble monomers can prime tau kinases under certain conditions, such as the activation of GSK-3β, CDK5, and p38 MAPK [54]. Soluble Aβ oligomers, particularly Aβ1–42 oligomers, are the most influential species in shaping tau pathology. These oligomers are highly synaptotoxic and directly interact with neuronal receptors, including PrPC, NMDAR, and β2-adrenergic receptors [55]. Aβ fibrils influence tau primarily indirectly; however, their structural properties are important. Aβ protofibrils expose hydrophobic surfaces that can template tau misfolding, enhancing tau nucleation more effectively than Aβ1–40 fibrils and affecting tau fibril architecture, contributing to tau strain divergence [56]. Aβ plaques serve as sources of multiple Aβ assemblies that influence tau, particularly those rich in oligomers, which correlate with early tau misfolding events or stimulate tau phosphorylation and seeding [57]. Significantly, the plaque microenvironment, which includes lipids, metal ions (Cu^2+^, Zn^2+^), and oxidative species, can alter Aβ–tau interactions and lead to region-specific tau filament folds [58].

Furthermore, Aβ promotes neuroinflammation and oxidative stress, both of which exacerbate tau pathology, as explained in the following paragraphs, thereby acting not only as a trigger but also as a facilitator of tau-mediated neurodegeneration [59]. Thus, the relationship between Aβ and tau is synergistic rather than linear [7], and therapeutic strategies should increasingly emphasize simultaneous modulation of both proteins.

Regarding other pathological changes in the neurodegenerated brain, neuroinflammation is now regarded as a major pathogenic component of tauopathies [60]. Microglia and astrocytes respond to pathological tau via pattern-recognition receptors, including TLR2, TLR4, and CX3CR1, thereby initiating cytokine release, inflammasome activation, and phagocytic responses [61]. Although initially protective, chronic activation becomes maladaptive and drives disease progression [62].

Microglia play a dual role in tau propagation. On the one hand, they attempt to clear extracellular tau aggregates [63]; on the other hand, they contribute to tau spread through exosome-mediated secretion of internalized tau seeds [64]. Studies demonstrate that microglial NLRP3 inflammasome activation accelerates tau phosphorylation, aggregation, and dissemination across neuronal networks [65]. Genetic deletion or pharmacological inhibition of NLRP3 reduces tau pathology and rescues cognitive deficits in experimental models [66]. Similarly, complement signaling, particularly via C1q and C3, amplifies microglial synaptic pruning, leading to synapse loss that is strongly correlated with tau burden [67].

In addition to microglia, astrocytes also contribute to tau-driven neuroinflammation. Reactive astrocytes exhibit impaired glutamate handling, altered metabolic support, and increased release of inflammatory mediators [68]. Tau aggregates accumulate within astrocytes in tauopathies, promoting astrocytic dysfunction and further amplifying neuronal vulnerability [69]. Astrocytes influence tau uptake and degradation through pathways involving endocytosis and autophagy–lysosomal processing [70]. Dysfunction of these pathways compromises tau clearance and aggravates tau-related pathology.

Cytokines such as IL-1β, TNF-α, and IL-6 exert direct effects on tau phosphorylation by activating kinases, including p38 MAPK and GSK-3β [71]. Chronic inflammation also alters splicing factors such as SRSF1 and Tra2β, potentially influencing tau isoform expression [72]. Thus, neuroinflammation does not merely accompany tau pathology but shapes it by modulating phosphorylation, misfolding, aggregation, and intercellular spread.

Similarly, oxidative stress is a significant mechanism linked to tau pathology and to the onset and progression of neurodegeneration [73]. Neurons are exceptionally vulnerable to reactive oxygen species (ROS) because of their high metabolic demands and limited regenerative capacity [74]. A growing body of research shows that pathological tau disrupts mitochondrial dynamics, impairs electron transport chain activity, and interferes with mitophagy, thereby amplifying ROS production [75].

Hyperphosphorylated tau destabilizes microtubules, impairs mitochondrial transport, and causes energy deficits at synapses [76]. Tau also interacts directly with mitochondrial proteins such as dynamin-related protein 1 (Drp1), promoting mitochondrial fragmentation [77]. These defects lead to reduced ATP levels, increased ROS generation, and release of pro-apoptotic factors. ROS, in turn, modify tau through oxidative modifications—including nitration, carbonylation, and crosslinking—that increase its aggregation propensity and reduce its affinity for microtubules [78]. Moreover, oxidative stress activates redox-sensitive kinases (e.g., JNK, p38 MAPK) that further phosphorylate tau, thereby establishing a vicious cycle [79]. Lipid peroxidation products, such as 4-hydroxynonenal (4-HNE), can modify tau or alter proteostasis systems, including the ubiquitin–proteasome system and autophagy, thereby compromising tau clearance [80]. Oxidative DNA damage also affects neuronal survival pathways, sensitizing cells to tau toxicity [81]. Importantly, oxidative stress is detectable early in AD and several tauopathies, suggesting that tau-mediated mitochondrial dysfunction may be an early contributor to neurodegeneration rather than a late consequence.

**Figure 4:**
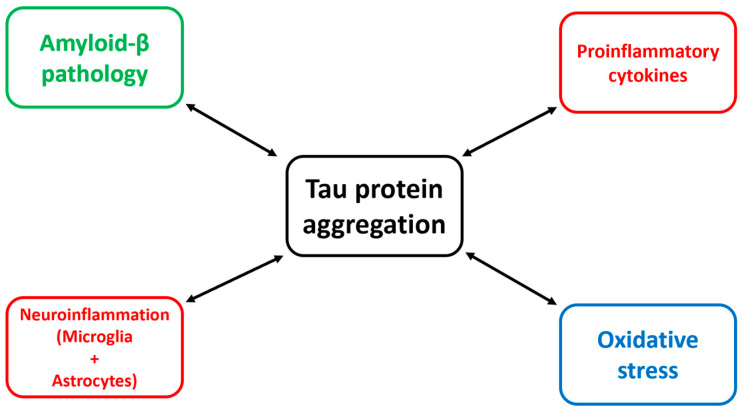
Schematic representation of the bidirectional relationship between tau pathology and other relevant neurodegenerative-related processes (green—amyloid pathology, red—inflammatory processes, blue—oxidative stress).

Table 2 and Figure 4 summarize the impact of tau pathology on other cellular and molecular changes observed in neurodegenerative disorders, showing that tau protein misfolding plays a central role in the incompletely understood neurodegenerative process.

## 3. Tau Protein Aggregation Inhibitors—Past, Present, and Future

Inhibiting tau protein aggregation is among the most direct therapeutic strategies to halt the core pathogenic process underlying tauopathies. Tau aggregation inhibitors aim to prevent the structural conversion of soluble tau into toxic oligomers and fibrillar assemblies [82]. Over the past two decades, multiple classes of tau aggregation inhibitors have been proposed, ranging from natural products such as curcumin to small-molecule compounds [83]. While most compounds remain in preclinical development, several have advanced into clinical trials, providing important insights into both the potential and the limitations of this strategy.

Phenothiazine derivatives were the first compounds demonstrated to inhibit tau aggregation in vitro and in vivo [84]. Among these, methylene blue (MB; methylthioninium chloride) emerged as the most extensively studied. MB inhibits tau aggregation by disrupting tau–tau interactions within the microtubule-binding repeat domain and destabilizing paired helical filaments [85]. Early preclinical studies demonstrated reduced tau pathology and improved cognitive performance in tau transgenic mouse models [86], although these models incompletely recapitulate the complexity and temporal progression of human tauopathies.

MB advanced to clinical evaluation under the trade name Rember™, with a Phase II trial in mild-to-moderate Alzheimer’s disease reporting dose-dependent cognitive stabilization [87]. However, limitations related to bioavailability, redox activity, and gastrointestinal side effects prompted the development of a reduced formulation, leuco-methylthioninium bis (LMTM; TRx0237) [88]. LMTM was evaluated in large Phase III trials in Alzheimer’s disease and behavioral variant frontotemporal dementia [89]. Despite promising preclinical data and post hoc analyses suggesting potential effects in monotherapy subgroups, these trials failed to demonstrate clear clinical benefit on the primary endpoints [89], raising concerns about statistical robustness, potential subgroup bias, and the translatability of tau-aggregation biomarkers into meaningful clinical endpoints. These results highlighted critical challenges, including trial design, disease stage selection, and the difficulty of translating aggregation inhibition into measurable clinical outcomes.

A wide range of natural polyphenolic compounds seems to exhibit tau anti-aggregation activity, often coupled with antioxidant and anti-inflammatory properties. Epigallocatechin-3-gallate (EGCG), a green tea catechin, has been shown to redirect tau aggregation toward off-pathway, non-toxic oligomers and to remodel preformed fibrils [90]. EGCG reduced tau pathology and neurotoxicity in cellular and animal models, although its limited brain bioavailability has constrained clinical translation [91].

Similarly, curcumin, resveratrol, myricetin, quercetin, and oleuropein aglycone showed tau fibrillization inhibitory properties by interfering with β-sheet stacking and stabilizing soluble tau conformations. Curcumin, a diarylheptanoid derived from *Curcuma longa*, inhibits tau fibrillization by binding to aggregation-prone regions within the microtubule-binding domain, thereby destabilizing β-sheet stacking and redirecting tau into soluble, non-fibrillar conformations [92]. In vitro studies show that curcumin can both prevent fibril formation and partially disaggregate preformed tau filaments, effects that are complemented by its antioxidant and anti-inflammatory properties [93]. In tau transgenic mouse models, curcumin reduces the burden of hyperphosphorylated tau and improves synaptic and cognitive outcomes [94]; however, clinical trials in Alzheimer’s disease have been largely inconclusive, primarily due to poor bioavailability, limited brain penetration, rapid systemic metabolism, and variability among curcumin formulations used in different studies, despite evidence of peripheral target engagement [95]. Resveratrol, a stilbene polyphenol found in grapes and red wine, interferes with tau aggregation by modulating β-sheet formation and indirectly affecting tau phosphorylation through activation of sirtuin-1 (SIRT1) and enhancement of proteasomal and autophagic degradation pathways [96]. Resveratrol promotes the clearance of misfolded tau species rather than acting as a direct fibril breaker [96]. In animal models, resveratrol reduces tau accumulation and attenuates neurodegeneration, particularly when administered at early disease stages [97], suggesting that therapeutic timing may critically influence efficacy in human disease. A Phase II clinical trial in mild-to-moderate Alzheimer’s disease demonstrated that resveratrol was safe and penetrated the blood–brain barrier, with biomarker changes suggestive of reduced neuroinflammation and altered Aβ and tau dynamics in CSF, although no significant cognitive benefit was observed [98], thus demonstration the challenge of translating modest biomarker modulation into clinically meaningful cognitive outcomes and raising questions about dose optimization and treatment duration.

Myricetin, a flavonol present in berries and tea, exhibits strong anti-aggregation activity by directly binding to tau repeat domains and inhibiting nucleation and elongation phases of fibril formation [99]. Structural studies indicate that myricetin disrupts hydrogen bonding within β-sheets and stabilizes tau in disordered conformations. In vitro, myricetin is more potent than many other flavonoids in preventing tau fibrillization and oligomer formation [100]. In vivo evidence remains limited, with few animal studies suggesting reduced tau pathology and oxidative stress, and no completed clinical trials specifically targeting tauopathies, reflecting challenges in formulation stability and bioavailability [101].

Quercetin, another widely distributed flavonoid, inhibits tau aggregation through a combination of direct and indirect mechanisms. Quercetin interferes with β-sheet assembly and reduces tau hyperphosphorylation by modulating kinase activity, including GSK-3β and CDK5, while also exerting antioxidant effects that limit oxidative modifications of tau [102]. Preclinical studies in cellular and rodent models demonstrate attenuation of tau aggregation and synaptic dysfunction [103]. Clinically, quercetin has been evaluated mainly for its antioxidant and anti-inflammatory effects in neurodegenerative and aging-related conditions; however, tau-specific clinical endpoints have not yet been systematically assessed [104].

Oleuropein aglycone, a phenolic compound derived from extra-virgin olive oil, inhibits tau aggregation by stabilizing monomeric tau and promoting the formation of non-toxic, off-pathway oligomers. It also enhances autophagic flux and proteostasis, facilitating the clearance of pathological tau species [105]. In transgenic mouse models of tauopathy and Alzheimer’s disease, oleuropein aglycone reduces tau and Aβ deposition, improves synaptic plasticity, and rescues cognitive deficits. Although direct clinical trials targeting tau pathology are lacking, epidemiological and interventional studies supporting the neuroprotective effects of the Mediterranean diet provide indirect clinical relevance [106].

While these compounds have demonstrated in vitro efficacy, their pleiotropic mechanisms, poor pharmacokinetics, and lack of target specificity have limited their progression beyond early-phase clinical or nutraceutical studies.

High-throughput screening efforts identified rhodanine and thiohydantoin derivatives as potent inhibitors of tau fibril formation. These compounds primarily act by blocking nucleation and elongation phases of aggregation. While effective in vitro, concerns regarding nonspecific reactivity and classification as pan-assay interference compounds (PAINS) have hindered their development [107].

Benzothiazole and benzimidazole derivatives, structurally related to amyloid-binding dyes, also inhibit tau aggregation by interacting with β-sheet-rich regions of tau fibrils. Some of these compounds overlap chemically with positron emission tomography (PET) tracers, underscoring shared structural motifs between imaging agents and aggregation inhibitors [108]. However, none have yet advanced to late-stage clinical trials.

Furthermore, newer drugs complement the previously described compounds and differ in their more precise, targeted mechanisms of action. Among this heterogeneous group of medications, notable are molecular tweezers, peptide-based inhibitors, and oligomer-targeting compounds. The molecular tweezer CLR01 represents a novel and highly specific approach to inhibiting tau aggregation. CLR01 selectively binds lysine residues via noncovalent supramolecular interactions, thereby preventing pathological protein–protein interactions while sparing physiological tau–microtubule binding [109]. CLR01 effectively inhibited tau oligomerization, reduced seeding activity, and ameliorated behavioral deficits in tau transgenic mouse models. Importantly, CLR01 also demonstrated activity against other amyloidogenic proteins, including Aβ and α-synuclein, suggesting potential utility in mixed-pathology neurodegenerative diseases [110]. While still preclinical, molecular tweezers are considered among the most promising next-generation tau aggregation inhibitors.

Peptide-based inhibitors targeting aggregation-prone hexapeptide motifs in tau, particularly PHF6 (VQIVYK) and PHF6* (VQIINK), have been extensively studied. Modified peptides, including N-methylated and β-sheet breaker variants, effectively block tau nucleation and fibril elongation in vitro [111]. However, challenges related to metabolic stability, blood–brain barrier penetration, and immunogenicity have limited their translational potential. More recently, structure-guided small molecules designed to bind disease-specific tau filament folds identified by cryo-electron microscopy have emerged as a promising avenue [112]. These compounds aim to achieve strain-selective inhibition, potentially addressing the heterogeneity observed across tauopathies. Although still in early discovery phases, this approach represents a paradigm shift toward precision targeting of tau aggregation.

Some molecules designed to target other pathological mechanisms exhibited unexpected interactions with tau. One relevant example is Anle138b, a small molecule initially developed as an α-synuclein aggregation inhibitor that later showed inhibition of tau oligomer formation [113]. Anle138b reduces toxic oligomer species without entirely preventing fibril formation, thereby targeting the most neurotoxic tau assemblies. In tau transgenic mouse models, anle138b improved synaptic function and reduced neurodegeneration [114]. The compound has entered early clinical development, positioning it among the most translationally advanced oligomer-directed tau inhibitors.

Despite preclinical success, many of the aforementioned tau aggregation inhibitors (summarized in Table 3) failed to demonstrate efficacy in subsequent clinical trials, resulting in disappointing outcomes. Significant challenges include insufficient brain exposure, the absence of biomarkers that directly reflect aggregation inhibition, late intervention in the disease course, and the possibility that aggregation inhibition alone may be insufficient once downstream neurodegenerative cascades are established [115].

In addition to small-molecule aggregation inhibitors, immunotherapeutic approaches targeting tau have emerged as a major therapeutic strategy in recent years. Both active and passive tau immunotherapies are designed to reduce pathological tau species, primarily by promoting extracellular clearance, limiting trans-synaptic propagation, or facilitating microglial-mediated degradation of pathological aggregates [116]. Several monoclonal antibodies, such as gosuranemab (BIIB092) [117], tilavonemab (ABBV-8E12) [118], semorinemab (RO7105705) [119], and zagotenemab (LY3303560) [120], have advanced to Phase II and III clinical trials in Alzheimer’s disease and primary tauopathies. Although these trials have generally demonstrated acceptable safety profiles, most have failed to meet primary cognitive or functional endpoints, highlighting challenges related to epitope selection, disease stage, intracellular target accessibility, and biomarker–clinical endpoint dissociation [116]. Importantly, tau antibodies primarily aim to neutralize extracellular or propagating tau species rather than directly inhibit intracellular aggregation processes. Therefore, while immunotherapy represents a complementary tau-directed strategy, it differs from aggregation inhibitors, which target early misfolding and fibrillization events. The limited clinical efficacy observed to date further underscores the need for combinatorial or multi-target approaches that simultaneously address tau aggregation, propagation, and amyloid pathology [116].

## 4. Perspectives on Dual Tau and Amyloid Beta Inhibitors

A valuable strategy for developing new anti-AD agents is the dual inhibition of Aβ and tau aggregation to reduce levels of both pathological peptides. Accumulating evidence indicates that Aβ oligomers accelerate tau hyperphosphorylation and aggregation, while tau is required for many of the synaptotoxic and pathogenic effects of Aβ. This interdependence suggests that interventions targeting a single pathogenic species may be insufficient; this is supported by the lack of effective disease-modifying therapies in AD. However, while conceptually attractive, dual-target approaches must balance enhanced therapeutic impact with increased molecular complexity and potential pharmacokinetic liabilities. Among the compounds described in the previous subchapter, curcumin and EGCG have also been shown to interfere with the aggregation of both pathological proteins and promote the disassembly of existing fibrils through combined hydrophobic and hydrogen-bonding interactions [121], although their pleiotropic mechanisms may complicate the identification of primary target engagement in vivo.

Other multitarget-directed ligands are represented by synthesized derivatives, such as 2,4-thiazolidinedione (TZD)–based bivalent derivatives developed to target both Aβ and tau aggregation simultaneously. According to Gandini et al., twenty-four compounds were synthesized and initially screened in intact Escherichia coli cells overexpressing Aβ42 and tau, enabling rapid assessment of dual anti-aggregation activity in a cellular context [122]. Lead compounds were subsequently evaluated for neuronal toxicity, blood–brain barrier permeability, and direct interactions with isolated Aβ42 and tau protein in vitro. Based on combined efficacy, safety, and BBB penetration, two candidates (compounds **22** and **23**) were advanced to in vivo testing in a *Drosophila melanogaster* AD model. Notably, the carbazole-containing derivative **22** significantly improved the lifespan and locomotor performance of Aβ42-expressing flies at 20 μM and reduced brain Aβ42 aggregate burden, outperforming doxycycline at a higher concentration [122]. These findings identify compound **22** as a promising dual Aβ/tau aggregation inhibitor and support further development of TZD-based bivalent molecules as potential disease-modifying agents for AD [122]. Still, validation in mammalian models and careful pharmacokinetic optimization will be essential to determine their translational feasibility and their potential clinical impact.

Another relevant example is the class of 1,2,3,4-tetrahydro-1-acridone analogues, which was rationally designed and synthesized as dual aggregation inhibitors targeting both Aβ and tau proteins. The library incorporated N-methylation of the quinolone ring, a modification that markedly enhanced inhibitory activity against protein aggregation. In vitro fluorescence assays demonstrated that selected analogues (compounds **25**–**30**) at 20 µM suppressed Aβ aggregation by 84.7–99.5% and tau aggregation by 71.2–101.8%, indicating potent inhibition of β-sheet formation in both proteins [123]. The introduced cationic charge and specific substitutions on the tetrahydroacridone scaffold were shown to enhance dual-target engagement. One representative compound, designated compound **30**, exhibited favorable blood–brain barrier permeability in predictive models and bound to both Aβ and tau, consistent with its ability to disrupt β-sheet assembly and tau misfolding in cellular assays [123]. Mechanistically, these derivatives are thought to interact via noncovalent binding to key aggregation-prone regions on both Aβ and tau, thereby destabilizing β-structure formation and preventing the transition to toxic oligomers and fibrils. However, the introduction of cationic features and extended aromatic structures may increase the risk of off-target interactions and nonspecific protein binding.

Furthermore, a series of multifunctional benzylamino-hydroxyalkyl derivatives was rationally designed as dual aggregation inhibitors targeting both Aβ and tau, while simultaneously modulating additional AD–relevant pathways. These compounds integrate structural motifs that interact with β-sheet-forming regions of Aβ and tau, as well as pharmacophores that confer cholinesterase (AChE and BuChE) and β-secretase (BACE1) inhibitory activity [124]. In vitro aggregation assays demonstrated that selected analogues inhibited Aβ and tau fibrillization at low micromolar concentrations, achieving a balanced suppression of β-sheet formation for both proteins. Structure–activity relationship analyses indicated that the benzylamino group promotes π–π stacking and hydrophobic interactions with amyloidogenic cores, whereas the hydroxyalkyl side chain provides conformational flexibility and hydrogen-bonding capacity, facilitating dual-target engagement [124]. Importantly, lead compounds exhibited nanomolar-to-micromolar inhibition of AChE and BuChE and reduced BACE1 activity, highlighting their potential to simultaneously attenuate amyloid production and aggregation, as well as cholinergic dysfunction. Several analogues showed favorable predicted blood–brain barrier permeability and low cytotoxicity in neuronal cell models. On the other hand, simultaneous modulation of multiple enzymatic systems may increase the probability of unintended pharmacodynamic effects and complicate dose optimization in clinical settings.

The design of dual anti-aggregation agents can also begin with natural compounds with strong anti-inflammatory and antioxidant properties. A good example is the curcumin scaffold, whose structure is included in a series of potential multifunctional ligands. Compound **PE859** is a representative example of such ligands, which was found to exhibit more potent dual anti-aggregating properties than the parent compound. Moreover, it crossed the BBB, improved memory in vivo, reduced the number of aggregated lesions in mouse brains, and exhibited a promising pharmacokinetic profile [125]. Still, long-term safety, metabolic stability, and reproducibility across disease stages remain to be fully established before clinical translation can be considered.

Dual Aβ- and tau-anti-aggregating properties were combined with anticholinesterase activity to achieve disease-modifying and symptomatic effects. However, it should be noted that these hybrid molecules were designed as dual-binding-site cholinesterase inhibitors with potential anti-aggregating properties against Aβ. The hybrids described by Muñoz-Torreo et al. were formed by fusing 6-chlorotacrine, a potent AChE inhibitor, to the previously described tetrahydrobenzonaphthyridine derivatives, which interact with both active sites of AChE and exhibit weak Aβ aggregation-inhibitory activity [126]. This combination enabled the identification of a set of compounds that target tau, Aβ, and cholinesterases. The most potent derivative inhibited hAChE with an IC50 value of 2.06 nM and hBuChE with 0.286 μM, as well as Aβ42 and tau aggregation by 77.5% and 68.7%, respectively, in recombinant Escherichia coli cells at a concentration of 10 μM. However, a significant disadvantage of this compound is its low drug-like properties. Similarly, a series of shogaol–huprine hybrids displaying antioxidant properties, in addition to anticholinesterase and anti-aggregating activities, was developed, which was characterized by poor drug-likeness.

## 5. Conclusions and Future Perspectives

In the existing literature, tau protein is established as a key molecule that plays significant roles in cytoskeletal organization, intracellular transport, synaptic signaling, and nuclear protection, becoming profoundly maladaptive when disrupted by disease-associated modifications. Developmentally regulated splicing, precise phosphorylation, and dynamic intracellular and extracellular trafficking are essential for normal tau function; however, dysregulation of these processes initiates a cascade of pathological events, including mislocalization, aggregation, liquid–liquid phase separation, and prion-like propagation. Tauopathies are defined not merely by tau accumulation but by distinct, disease-specific filament folds and isoform compositions shaped by post-translational modifications and interacting pathologies. Tau pathology does not act in isolation to produce and sustain neurodegeneration; instead, it is embedded in a self-reinforcing network involving amyloid-β, neuroinflammation, mitochondrial dysfunction, and oxidative stress, each of which amplifies tau toxicity and spread. These interdependencies help explain clinical heterogeneity, progressive neurodegeneration, and the limited efficacy of single-target interventions. In this context, effective disease-modifying therapies for AD and related tauopathies require strategies that address tau aggregation and propagation while simultaneously modulating upstream drivers and downstream consequences, including neuronal activity, inflammatory signaling, and cellular proteostasis.

Efforts to inhibit tau aggregation have provided a critical proof of concept that directly targeting the structural conversion of tau is biologically feasible and can attenuate pathology in experimental models. However, the limited clinical success of first-generation compounds, such as methylene blue and its derivatives, underscores the challenges posed by inadequate brain exposure, pleiotropic pharmacology, and suboptimal trial design. Natural polyphenols and early small-molecule inhibitors are important for revealing key mechanistic principles, such as redirecting tau into off-pathway assemblies or enhancing proteostatic clearance. Still, their poor specificity and pharmacokinetic limitations have constrained clinical translation. More recent strategies, including molecular tweezers, oligomer-selective inhibitors, peptide-based approaches, and structure-guided, strain-selective compounds with dual-targeting properties, represent a conceptual shift toward precision modulation of the most toxic tau species. Collectively, these advances suggest that future therapeutic success will depend on improved engagement of brain targets, early intervention, and selective inhibition of pathogenic tau conformers rather than global suppression of aggregation, potentially in combination with complementary approaches that address upstream and downstream disease mechanisms.

## Figures and Tables

**Table 1:** The roles of tau protein in physiological conditions.

Targeted Structure/Function	Physiological Role of Tau	Key Mechanism	Relevant References
Axonal microtubules	Microtubule stabilization and assembly	3R/4R microtubule-binding repeats interact with tubulin; affinity regulated by site-specific phosphorylation	[20,21]
Neuronal development	Neurite outgrowth and axonal maturation	Developmentally regulated MAPT expression and phosphorylation; predominance of 0N3R isoform in early brain	[22]
Actin cytoskeleton	Actin bundling and cytoskeletal cross-linking	Multivalent interactions of proline-rich and microtubule-binding domains with filamentous actin	[24]
Synaptic structure and signaling	Maintenance of dendritic structure and synaptic signaling	Interaction with Fyn kinase via N-terminal domain; modulation of NMDA receptor signaling	[23,25]
Myelination	Support of process outgrowth and axonal myelination	Tau–Fyn signaling axis in oligodendrocytes	[25]
Isoform diversity	Functional and developmental adaptation	Alternative splicing of MAPT exons 2, 3, and 10, generating 0N/1N/2N and 3R/4R isoforms	[21]
Nucleus stability	DNA binding and genome protection	Direct interaction with DNA contributes to genomic stability	[26]
Extracellular compartment	Biomarker of neuronal activity and turnover	Release of soluble tau into interstitial fluid and cerebrospinal fluid	[27]

**Table 2:** The impact of tau pathology on other relevant neurodegenerative-related processes.

Pathological Processes	Tau-Related Mechanisms	Impact on Neurodegeneration	Relevant References
Amyloid-β pathology	Aβ oligomers promote tau hyperphosphorylation and mislocalization; tau mediates Aβ synaptotoxicity via Fyn–NMDA signaling	Synaptic dysfunction, impaired axonal transport, enhanced tau aggregation, and spread	[48,49,50,51,52]
Aβ-driven tau strain diversity	Aβ species influence tau filament conformation and folding environment	Disease-specific tau conformers and clinical heterogeneity in AD	[55,56,57,58]
Neuroinflammation (microglia)	Tau activates PRRs/NLRP3 inflammasome; microglial uptake and exosomal tau release	Chronic inflammation, increased tau propagation, and synaptic loss	[63,64,65,66,67]
Neuroinflammation (astrocytes)	Astrocytic tau accumulation and impaired lysosomal clearance	Glutamate dysregulation, reduced neuronal support, amplified tau toxicity	[68,69,70]
Cytokine signaling	Proinflammatory cytokines (IL-1β, TNF-α, IL-6) activate tau kinases (GSK-3β, p38 MAPK)	Sustained tau hyperphosphorylation and aggregation	[71,72]
Oxidative stress	Tau-associated mitochondrial dysfunction increases ROS; oxidative modifications of tau	Feed-forward tau aggregation, reduced microtubule binding, neuronal energy failure	[73,74,75]

**Table 3:** An overview of tau aggregation inhibitors—major classes, mechanisms of action, and relevant trials.

Major Class	Representative Compound(s)	Mechanism(s) of Action	Preclinical Findings	Relevant Clinical Trials/Development Status
Phenothiazine derivatives	Methylene blue (MB; Rember™) Leuco-methylthioninium bis (LMTM; TRx0237)	Disrupt tau–tau interactions; destabilize PHFs	Reduced tau pathology and improved cognition in tau mice	MB: Phase II AD (dose-dependent cognitive stabilization) LMTM: Phase III AD and bvFTD—no primary endpoint benefit
Natural polyphenols (Catechins)	EGCG (green tea)	Remodel fibrils; redirect aggregation to non-toxic species	Reduced tau pathology in cellular and animal models	No advanced tau-focused clinical trials (poor brain bioavailability)
Natural polyphenols (Diarylheptanoids)	Curcumin	Binds MTBR; inhibits β-sheet formation	Reduced hyperphosphorylated tau; improved cognition in mice	AD clinical trials inconclusive (poor bioavailability)
Natural polyphenols (Stilbenes)	Resveratrol	Indirect anti-aggregation via SIRT1 activation; enhances proteostasis	Reduced tau accumulation in animal models	Phase II AD: BBB penetration and biomarker effects; no cognitive benefit
Natural polyphenols (Flavonols/flavonoids)	Myricetin, quercetin	Direct binding to repeat domains; inhibit nucleation/elongation; kinase modulation	Strong in vitro inhibition; limited in vivo data	No completed tau-specific clinical trials
Natural polyphenols (Phenolic secoiridoids)	Oleuropein aglycone	Stabilizes monomeric tau; promotes off-pathway aggregation; enhances autophagy	Reduced tau/Aβ deposition; improved cognition in mice	No direct tau trials; indirect epidemiological support
Small-molecule PAINS	Rhodanine Thiohydantoin derivatives	Inhibit fibril nucleation/elongation (in vitro)	Strong in vitro inhibition	Development limited by nonspecific reactivity
Amyloid-binding dye–related compounds	Benzothiazole, benzimidazole derivatives	Bind β-sheet-rich fibrillar regions	Inhibit tau aggregation in vitro	No late-stage clinical trials
Molecular tweezers	CLR01	Selective lysine binding; disrupt pathological protein interactions	Reduced oligomerization and behavioral deficits in mice	Preclinical data only
Peptide-based inhibitors	PHF6/PHF6*–targeting peptides	Block aggregation at hexapeptide motifs	Strong in vitro inhibition	Mostly preclinical data Translational challenges
Structure-guided, strain-selective inhibitors	Cryo-EM-guided small molecules	Bind disease-specific tau folds; strain-selective inhibition	In early discovery phases	In early discovery phases
Oligomer-targeting small molecules	Anle138b	Preferentially reduces toxic tau oligomers	Improved synaptic function and reduced neurodegeneration in mice	Early clinical development

## Data Availability

No new data were created or analyzed in this study.

## Abbreviations

The following abbreviations are used in this manuscript:

AD	Alzheimer’s Disease
Aβ	Amyloid beta
CBD	Corticobasal Degeneration
CDK5	Cyclin-Dependent Kinase 5
CSF	Cerebrospinal fluid
CX3CR1	C-X3-C Motif Chemokine Receptor 1
EGCG	Epigallocatechin Gallate
FTLD	Frontotemporal Lobar Degeneration
GSK3β	Glycogen Synthase Kinase 3 Beta
IL-1β	Interleukin-1 beta
LMTM	Hydromethylthionine
MAPT	Microtubule-Associated Protein Tau
NFT	Neurofibrillary Tangle
NMDA	N-Methyl-D-Aspartate
PAINS	Pan-Assay Interference Compounds
PET	Positron Emission Tomography
PSP	Progressive Supranuclear Palsy
PTM	Post-Translational Modification
ROS	Reactive Oxygen Species
SIRT1	Sirtuin 1
TLR	Toll-like Receptor
TNF-α	Tumor necrosis factor alpha
